# Exploring the effects of dietary lysine and tryptophan on the social behavior of pigs

**DOI:** 10.1093/jas/skaf030

**Published:** 2025-02-06

**Authors:** Eleanor Hewett, Luis Zaragoza, Craig Lewis, Jos Houdijk, Andrea Wilson, Simon Turner

**Affiliations:** Animal Behaviour and Welfare, Animal and Veterinary Sciences Department, Scotland’s Rural College (SRUC), Edinburgh EH9 3JG, UK; Global Research and Communication Manager, Pig Improvement Company (PIC), Hendersonville, TN, USA37075; PIC Product Development PIC, C/Pau Vila, Barcelona, Spain; Monogastric Science Research Centre, Agriculture and Land-based Engineering Department, Scotland’s Rural College (SRUC), Edinburgh, EH9 3JG, UK; The Roslin Institute, The Royal (Dick) School of Veterinary Studies, The University of Edinburgh, Edinburgh, EH25 9RG, UK; Animal Behaviour and Welfare, Animal and Veterinary Sciences Department, Scotland’s Rural College (SRUC), Edinburgh, EH9 3JG, UK

**Keywords:** behavior, lysine, pig, tryptophan, welfare

## Abstract

Negative social behaviors between pigs can cause stress, which can compromise welfare. There has been significant interest in exploring the effect of diet on negative social behaviors and the wider social behavior repertoire of pigs. The aim of this study was to determine the effects of dietary Lysine (Lys) and Tryptophan (Trp) levels on the social behavior of commercially housed pigs. A total of 2,293 PIC Camborough barrows with a mean starting weight of 11.87 ± 1.35 kg were used in a randomized complete block design with a 2 × 3 factorial arrangement, with 16 replicates per treatment, of the following factors: 1) Standardized ileal digestible Lys levels: 100% Lys = diets with 100% PIC requirement at the midpoint of the growth phase (Lys 100) vs 80% Lys = diets with 80% PIC requirement at the midpoint of the growth phase (Lys 80); and 2) Trp to Lys ratio of 0.210, 0.185, or 0.160. Pigs were randomly allocated across the 6 treatments over 2 starting dates. Behavior and lesion data were collected. There was an effect of Lys (*P* = 0.032) on ear-biting behavior, with pigs on the Lys 80 treatments showing a higher level of ear-biting behavior. We also found an effect of the Lys score week interaction on the proportion of pens showing ear lesions (*P* < 0.001) and an effect of the Lys Trp interaction (*P* = 0.030) and the Lys score week interaction (*P* = 0.0104) on the proportion of pens showing severe ear lesions. In conclusion, the lysine content of feed can affect the social behavior of pigs, specifically ear biting, in commercial conditions.

## Teaser

Negative social behaviors continue to be an issue for intensively farmed pigs. Manipulating the lysine and tryptophan content of pig feed could help to reduce negative social behaviors, with a possible positive effect on production measures.

## Introduction

Pigs (Sus scrofa) are highly social animals with a complex repertoire of social behaviors. However, intensive farming systems (often characterized by a small space allowance; Lammers and Schouten, 1985), unstimulating environment (Cox and Cooper, 2001), and competitive feeding systems (Day et al., 1995), impact the behavior of pigs and contribute to a divergence from social behaviors seen in the wild (Stolba and Wood-Gush, 1989). Negative social behaviors (as defined for this paper as behaviors that involve more than one pig where one of the pigs involved shows an avoidant or aggressive response on receipt of said behavior, e.g., tail biting, ear biting, flank biting, fighting) are more commonly seen in intensive farm systems (Taylor et al., 2010). Other naturally performed, positive social behaviors are shown less commonly e.g., play (Newberry et al., 1988). Aggressive encounters induce a stress response in pigs (Marchant et al., 1995) and often result in skin lesions that are likely to be painful (Di Giminiani et al., 2017) and which are open to infection. Ear, tail, and flank biting can lead to significant lesions, necrosis and loss of tissue, infection, and abscess formation (Sihvo et al., 2012) and in the most severe cases can result in death or euthanasia of the pig or condemnation of the carcass at slaughter. Play behavior has been proposed as an indicator of high animal welfare, as research suggests that play is more commonly displayed under good environmental conditions (Lawrence, 1987; Lawrence et al., 2018). Studies have also shown that play behavior positively relates to weight gain both pre and postweaning (Brown et al., 2015; Franchi et al., 2023). It therefore benefits both the pig and the producer to reduce negative social behaviors and encourage positive social behaviors.

Numerous solutions to reduce negative social behaviors have been proposed, including reducing stocking density (Cho and Kim, 2011), providing enrichment (van de Weerd and Day, 2009), increasing early life socialization (Weller et al., 2019), and reducing regrouping (Camerlink et al., 2021). However, many of these strategies have economic and space constraints and are difficult to implement in current intensive farming systems, and are therefore not widely adopted (Peden et al., 2021). Consequently, we are still searching for an economically viable and commercially relevant strategy to reduce negative and promote positive, social behaviors in pigs. One area that deserves further investigation is nutrition. Literature suggests that nutrition and the feeding system may have important impacts on the behavior and welfare of pigs, including fiber content (Bakare et al., 2014; Chou et al., 2020), protein content (McAuley et al., 2022), amino acid composition (Meer et al., 2017; Minussi et al., 2023), vitamin and mineral content, e.g., magnesium and salt (Jankevicius and Widowski, 2004) and vitamin B6 (Castilha et al., 2016), but also feeding system factors including ad libitum vs restricted feeding (D’Eath et al., 2009), wet vs dry feeding (Andersen et al., 2023) and pellet size (Brand et al., 2014).

There is increasing interest in exploring the effects of the amino acid composition of diets on the negative social behaviors of pigs, particularly lysine (Lys) and tryptophan (Trp). Lys is the first limiting amino acid in the majority of pig diets, determining growth and lean muscle deposition (Main et al., 2008). Trp is important for protein synthesis and is the primary precursor of serotonin (5-hydroxytryptamine, 5-HT) and melatonin. Trp and subsequently serotonin play major roles in regulating behavioral and physiological processes in pigs such as feed intake (Baranyiová, 1991), mood (Markus et al., 2000), and stress hormone secretion (Adeola and Ball, 1992).

There is already a small body of research focusing on the effect of Trp on social behavior in pigs (Meunier-Salaün et al., 1991; Koopmans et al., 2005, 2006, 2012; Li et al., 2006, 2011; Shen et al., 2012a, 2012b; Liu et al., 2013; Poletto et al., 2014, 2014; Castilha et al., 2016; Stracke et al., 2017; Lay et al., 2021; Henry et al., 2022). However, the studies administered Trp at levels several fold higher than the commercial norm in pig diets, which is likely to be prohibitively costly in commercial production systems. Studies are lacking which investigate the effect of Trp on social behavior within the range commonly used in industry across the United States and Europe and no study has given attention to the effect on positive social behaviors. Furthermore, despite being the first limiting amino acid, only one study has explored the effect of increasing Lys levels on social behavior (Minussi et al., 2023).

The aim of this study was to explore the effect of Lys and Trp on both positive and negative social behaviors of pigs, at levels modestly above current commercial levels. We hypothesized that pigs on high Lys diets would show reduced levels of negative social behaviors and increased levels of positive social behaviors. We also hypothesized that for pigs on diets low in Lys but high in Trp, the Trp would mitigate the effect of the low Lys and those pigs would not show the increase in negative social behaviors.

## Materials and Methods

### Ethical review

The study was approved by the Scotland’s Rural College (SRUC) Animal Experiments Committee under application PIG AE 22-2022. Routine animal care and management were performed by trained facility staff.

### Animals and housing

Behavioral observations were carried out on an initial cohort of 2,293 PIC Camborough barrows, mean starting weight of 11.87 ± 1.35 kg. Pigs were housed under U.S. commercial conditions (no enrichment), in a fully slatted house, consisting of 2, tunnel ventilated barns. Each barn contained 48 pens of 2.25 × 2.25 m (with 2 additional smaller pens per barn which were used as the intensive care pens) with an initial inventory of 16 to 26 castrated male pigs per pen. Pens were fitted with one feeder, divided into 3 sections and 2 pen-mounted bowl drinkers. Pigs arrived on farm on the 8th, 11th, and 15th of August after weaning at a separate facility. Barn 1 pigs were put on treatment on the 30th of August and barn 2 on the 8th of September. Behavioral observations were taken over a period of 9 wk from the 10th of October to 11th of December.

### Experimental treatments

Pigs were randomly allocated across 6 treatments in a 2 × 3 factorial arrangement in a randomized complete block design (block = wean date and weight), giving 16 replicates of each treatment, 8 per barn, of the following iso-energetic feeding treatments: 1) Standardized ileal digestible (SID) Lys levels: Lys 100 = diets with 100% PIC requirement at the midpoint of the growth phase, vs Lys 80 = diets with 80% PIC requirement at the midpoint of the growth phase; and 2) Trp to Lys ratio (Trp:Lys) of 0.210, 0.185, or 0.160. Full diet compositions, which had a basis of corn, distiller’s dried grains with solubles, and soybean meal, can be found in Appendix 1. Targeted Lys levels were attained through replacing soybean meal and synthetic Lys with corn, and minor modification from synthetic amino acid sources, minerals, and oil to ensure similar ratios of other essential amino acids to Lys at SID level, same levels of net energy, calcium, and standardized total tract digestible phosphorus as per recommendations of the genetic supplier (PIC, 2016) and (NRC, 2012) requirements. Within those Lys levels, targeted levels of Trp were attained by iso-energetic exchange L-tryptophan against a combination of corn and corn oil. The SID Lys levels were derived from a meta-analysis performed by PIC to determine the SID Lys requirements for the average requirement of average daily gain and feed-to-gain ratio for finishing PIC Camborough pigs under commercial conditions (Goncalves et al., 2017).

There were a total of 5 feeding phases, pigs were weighed at the end of each feeding phase. Feed delivery was recorded automatically by the feed system and feed remaining in the feeders at the end of the feeding phase was recorded to allow for calculation of feed consumption. Feed samples were taken by the research staff.

In discussion with veterinarians, pigs on the Lys 80 with Trp:Lys 0.160 treatment were moved to the Lys 100 with Trp:Lys 0.185 diet at the end of feeding phase 2 due to an excessive number of pens reaching the production and/or welfare endpoints, which were defined prior to the trial as the point at which a pigs growth rate was lagging more than 20% behind that of their counterparts on a standard diet or 2 animals required treatment or removal from the pen within a 7-d period of each other due to injury from social interactions.

### Behavior observations

Behavior observations were taken live by a single observer and via digital recordings collected using a GoPro HERO10 Black (GoPro Incorporated, San Mateo, CA, US). Digital recordings were scored by the same observer as the live recordings after the trial had ended. Data from both the live observations and observations taken from recordings were used in the dataset. Observations were taken between the hours of 08:00 and 19:00 over a period of 9 wk from the 10th of October to the 11th of December. The order of pen observations was determined in accordance with a Latin square design to reduce the impact of observation order on results. Each pen was observed 5 times over a daily period, whereby each pen was observed twice in the morning and 3 times in the afternoon (to avoid time periods when pigs were most likely to be sleeping) for 15 min each, giving a total of 75 min per day. Each pen was observed on 3 observation days, twice live and once by video recording.

Prior to the commencement of the observation, the observer entered the pen to disturb the pigs. After all pigs had been roused to standing, the observer exited the pen. On exiting the pen, the observer waited 2 min for the pigs to settle down and resume their undisturbed behavior. This process was carried out at the beginning of both live and recorded observation periods.

Behaviors of all pigs (live and recorded) were recorded using an ethogram (Table 1), using a combination of continuous observation (for short-lived behaviors) and scan sampling (for posture). For the continuous observations the frequency but not duration of behaviors was recorded. At time 0 and at intervals of 150 s after, a tally was taken by scan sampling of the number of animals lying, sitting, standing, eating, or drinking, each either alone or with another animal.

**Table 1.** Ethogram used to record behaviors of pigs in both live and recorded observations.

Behavior	Description	Reference
**Scan sampling**		
Lying together	A pig is resting, with eyes closed or opened, with the body in full contact with the floor while at least a quarter of their body is in contact with another lying pig	(Camerlink et al., 2021)
Lying alone	A pig is resting, with eyes closed or open, with the body in full contact with the floor while less than a quarter of its body is in contact with another lying pig	Adapted from (Camerlink et al., 2021)
Sitting together	Pig is sitting with back legs and rump in contact with the floor, resting on or touching another pig	Defined for this study
Sitting alone	Pig is sitting with back legs and rump in contact with the floor, not resting on or touching another pig	Defined for this study
Standing together	A pig is standing, sniffing, touching, or rooting the surroundings (e.g., floor or wall) while the front part (front shoulder to tip of nose) or all of its torso is touching another pig.	Adapted from (Camerlink et al., 2021)
Standing alone	A pig is sniffing, touching, or rooting the surroundings (e.g., floor or wall) while no part of its front body region (defined above), or less than 100% of its torso, is touching another pig	Adapted from (Camerlink et al., 2021)
Eating/drinking together	Two or more pigs are engaged in eating or drinking at the same time from adjacent feeders/drinkers	Defined for this study
Eating/drinking alone	Single pig is engaged in eating or drinking	Defined for this study
**Continuous sampling**		
Social nosing	Non-agonistic nosing, including allogrooming and gentle nose touching. Behavior does not escalate to aggressive behavior or elicits evasive action from recipient pig	Adapted from (Camerlink et al., 2021)
Social play	A pig is scampering, running, pivoting, head tossing, flopping, hopping nudging, pushing, climbing, or non-harmful fighting with at least one other pig. All pigs involved in social play show at least one of these play markers.	(Blackshaw et al., 1997; Brown et al., 2015; Camerlink et al., 2021)
Individual play	A pig is scampering, running, pivoting, head tossing, flopping, or hopping alone.	(Newberry et al., 1988; Donaldson et al., 2002; Brown et al., 2015)
Aggression	Head knocking, fighting, biting, ramming	Adapted from (Camerlink and Turner, 2013)
Mounting	Standing on hind legs while having front legs on other pig’s body	(Camerlink and Turner, 2013)
Ear/ tail/flank biting	Taking the ear or tail of a pen mate into the mouth or nibbling, sucking or chewing the ear, tail or body of a pen mate	Adapted from (Camerlink and Turner, 2013)
Belly nosing	Repetitive up and down snout movement on the belly of a pen mate	(Camerlink and Turner, 2013)

Observations were stopped if pigs were disturbed during the 15-min observation period and some observation periods were discounted due to poor video quality. A total of 3.40% of observations could not be carried out.

### Lesion scoring

Two different types of lesion scoring were performed, one to capture lesions caused by biting of the tail, ears, and flank and the other to capture lesions caused by fighting/aggressive behaviors.

Bite lesions were scored weekly for 8 wk for a total of 7 recording events (1 wk it was not possible to take lesion recordings as it was deemed unsafe for the observer to enter the pens after a feeder outage). Once a week, all pens were examined and all pigs were scored for damage and freshness of ear, tail, and flank bites (Table 2). The observer entered the pen and moved slowly through the pigs, ensuring all pigs were disturbed in order to observe as much of the pig as possible.

**Table 2.** Score system used for scoring ear, tail, and flank bites, both damage and freshness

Damage	Freshness
0- no evidence of damage to the skin	0- no evidence of damage to the skin
1—mild swelling, redness, and evidence of bite marks, broken skin	1—no blood
2- mild to severe swelling, redness, bite marks, broken skin, scabbing with some fresh blood	2—dry, black blood, scar formation
3—severe swelling, redness, open wound, fresh blood, necrosis of tissue	3—dark red blood, scabbing and some healing seen
4—more than 50% of the pig’s ear is missing/ whole tail is missing and stump has become an open wound, fresh blood and necrotic tissue	4—bright red, fresh blood

**Figure 1.**
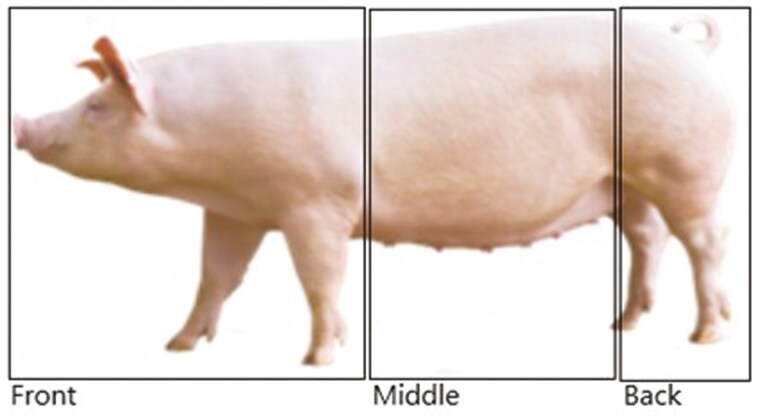
Body zones of the pig. Image taken from PIC, showing the Camborough pig.

Fight lesion scores were collected every other week over the 8-wk period for a total of 4 recording events. All pens were examined and fight lesions were scored on 6 pigs per pen. On entering the pen, the observer randomly selected and scored 2 large pigs, 2 mid-sized pigs, and 2 small pigs. The score (Table 3) and location of lesions were recorded. Location was determined by zone: front (tip of nose to back of shoulder of font legs), middle (back of the front shoulder to front of back legs), back (front of back leg to tip of tail; Figure 1). A lesion was defined as any continuous mark on the skin of the pig, longer than 1cm. Any other skin damage e.g., ulcers, heat rash, grazes, bruises, was noted.

**Table 3.** Lesion score system used to score fight lesions

Number of lesions	Score
0	0
1 to 10	1
11 to 20	2
21 to 30	3
31 to 40	4
41 to 50	5
51+	6

## Statistical Analysis

Statistical analyses were carried out using R Software version 4.2.2 (R Core Team, 2021). Models were constructed using the lme4 package (Bates et al., 2015). Pen was the experimental unit. Where appropriate, model residuals were visually inspected to confirm approximation to normality and tested for heteroscedasticity using Levene’s Test. When main effects (Lys or Trp:Lys) or interactions were not significant at *P* < 0.05, they were removed from the model. *P*-values below 0.05 were considered statistically significant and *P*-values between 0.05 and 0.1 were considered as tendencies.

### Behavior observations

Five treatments remained after moving pigs from treatment Lys 80 with Trp:Lys 0.160 to treatment Lys 100 with Trp:Lys 0.185. Behavioral data were not analyzed for these pigs.

Behavior scores for each pen were combined across observations and divided by the total number of pigs in the pen over the 3 observation days to give one frequency for each behavior per pen. For posture behaviors, scores were combined together and apart as they were highly correlated. Values were standardized to a mean of zero and standard deviation of 1 and data from pens on treatments Lys 100 with Trp:Lys 0.210, Lys 100 with Trp:Lys 0.185, Lys 100 with Trp:Lys 0.160, Lys 80 with Trp:Lys 0.210 and Lys 80 with Trp:Lys 0.185 were analyzed using principal component analysis. The principal component analysis identified 2 principal components (PC); PC1 accounted for 31% of the variation and PC2 accounted for 15%. The labels for the components were determined by selecting the highest loading descriptors for the positive and negative poles. Therefore, PC1 was labeled as activity (0.45 for standing, −0.46 for lying) and PC2 was labeled as social behavior (0.37 for ear biting, -0.60 for social play).

PC1 and PC2 were analyzed using a linear mixed model with ‘fill week’,’Ly’s, ‘Trp:Lys’ and the ‘Lys × Trp:Lys’ interaction as fixed effects and block as a random effect. Some select behaviors of interest (aggression, a combined play score [sum of individual and social play], and ear-biting behavior) were also analyzed as response variables using linear mixed models. ‘Fill week’, ‘Lys’, ‘Trp:Lys’ and the ‘Lys × Trp:Lys’ interaction were included as fixed effects. ‘Barn’ was included as a random effect.

### Lesions scores

For both the lesion score data sets, data from all 6 treatments was analyzed. For treatment Lys 80 with Trp:Lys 0.160, only data collected from pigs when they were on the treatment Lys 80 with Trp:Lys 0.160 diet was used (feeding phase 1 and 2). Lesions scores were divided by the number of pigs per pen to give a score based on lesions per pig.

### Bite lesions

For the ear, tail, and flank bite analysis, only ear bites occurred frequently enough for analysis. Pens were sorted into a binary data set; pens with evidence of ear biting and pens without evidence of ear biting. A second data set for pigs showing severe ear lesions (scores 2, 3, and 4) and all other pens was also established. The binary data set was analyzed using a generalized linear mixed model, with binomial distribution. Fixed effects were ‘Lys’, ‘Trp:Lys’, ‘Lys × Trp:Lys’ interaction, ‘score week’, ‘fill date’, and ‘barn’. Random effects were ‘block’ and ‘pen’, with ‘pen’ nested in ‘block’.

### Fight lesions

Fight lesion scores were also sorted into a binary data set; pens with scores above and pens with a score below the median score. The binary data set was analyzed using a generalized linear mixed model, with binomial distribution. Fixed effects were ‘Lys’, ‘Trp:Lys’, ‘Lys × Trp:Lys’ interaction, ‘score week’, ‘fill date’ ‘barn’, and ‘pig size’. Random effects were ‘block’ and ‘pen’, with ‘pen’ nested in ‘block’.

## Results

### Morbidity and mortality

Over the 9 wk of behavioral observations, 110 pigs were removed from the trial due to morbidity (*n* = 66) and mortality (*n* = 44). Date, weight, and suspected reason for death/removal were recorded where possible. The highest proportion of removals came from treatment Lys 80 with Trp:Lys 0.160 (33.64%) and the lowest proportion from treatment Lys 100 with Trp;Lys 0.210 (5.45%; Table 4). An additional 11 pigs died during the 9-wk observation period after being moved to the intensive care pens. As pigs were not individually identifiable, it was not possible to determine which treatment these 11 pigs had been on before moving to the intensive care pen.

**Table 4.** Number of pigs removed due to death and injury/illness from each dietary treatment

Treatment	Death	Injury/Illness	Total removed	%
Lys 100 with Trp:Lys 0.210	4	2	6	5.45
Lys 100 with Trp:Lys 0.185	8	4	12	10.91
Lys 100 with Trp:Lys 0.160	10	7	17	15.45
Lys 80 with Trp:Lys 0.210	6	9	15	13.64
Lys 80 with Trp:Lys 0.185	9	14	23	20.91
Lys 80 with Trp:Lys 0.160	7	30	37	33.64
Totals	44	66	110	100.00

### Behaviors

There was an effect of fill week (which corresponds to the pigs age) on PC1 (activity; *P* < 0.001). The average PC1 (activity) score for pigs of fill week 3 was higher than the average PC1 (activity) score for pigs of fill week 1. The effect of Lys tended towards significant (*P* = 0.066; being higher at Lys 80) but there was no effect of Trp:Lys on PC1 (activity). We found no effect of Lys or Trp:Lys on PC2 (social behavior). However, there was an effect of barn (*P* < 0.001) on PC2 (social behavior).

For the individual behaviors of interest, there was an effect of Lys (*P* = 0.032) and barn (*P* = 0.009) on ear-biting behavior. Pigs on lower Lys diets, Lys 80, showed higher levels of ear biting behavior and pigs in barn 2 showed more ear biting behavior than the pigs in barn 1. We found no effect of Lys, Trp:Lys, or the Lys × Trp:Lys interaction on aggressive behavior and only an effect of fill date on the combined play behavior (*P* = 0.034).

### Bite lesions

#### All ear lesions

**Figure 2.**
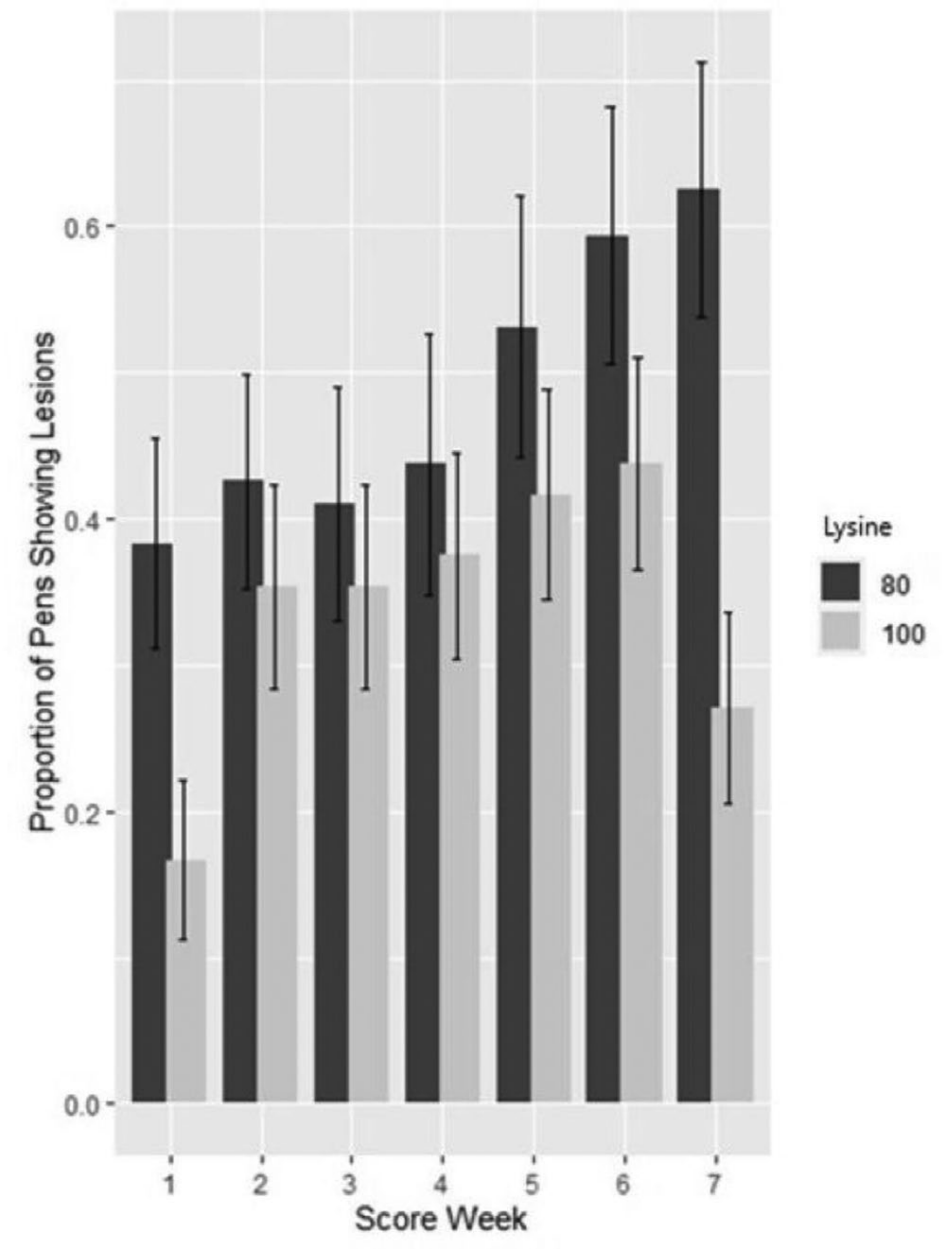
Effect of the interaction between lysine (Lys) level and score week (i.e., age) on the number of pens showing ear lesions (*P* < 0.001). The proportion of pens showing lesions, plotted against score week, grouped by lysine level; 100% Lys = diets with 100% PIC requirement at the midpoint of the growth phase (Lys 100) vs 80% Lys = diets with 80% PIC requirement at the midpoint of the growth phase (Lys 80). Forty-eight replicates per Lys level.

Lys and score week (i.e., age) interacted with the number of pens showing ear lesions (*P* < 0.001). At score weeks 1 and 7, the proportion of pens showing ear lesions was lower for pigs on the Lys 100 diet compared to pigs on the Lys 80 diet. For pigs on the Lys 80 diet, the proportion of pens showing lesions was higher in score week 7 than in score week 1 (Figure 2). We found no effect of fill date, barn, Trp:Lys or the Lys × Trp:Lys interaction on the number of pens showing ear lesions.

### Severe ear lesions

**Figure 3.**
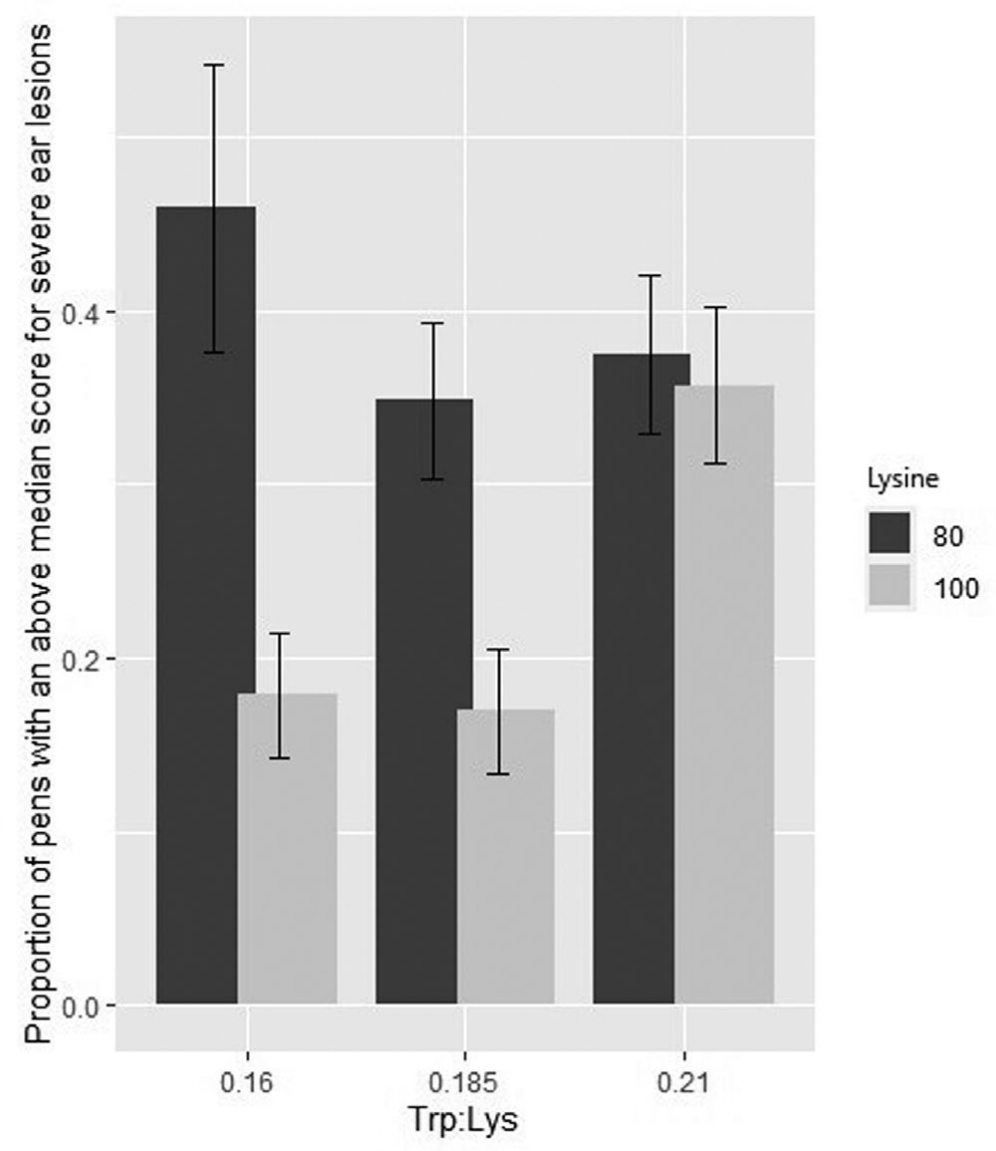
Effect of lysine (Lys) level and tryptophan to lysine ratio (Trp:Lys) interaction (*P* = 0.030) on the proportion of pens with an above-median score for severe ear lesions over an 8-wk period. 100% Lys = diets with 100% PIC requirement at the midpoint of the growth phase (Lys 100) vs 80% Lys = diets with 80% PIC requirement at the midpoint of the growth phase (Lys 80); and 2) Trp to Lys ratio of 0.210, 0.185, or 0.160. 48 replicates per Lys level, 32 replicates per Trp:Lys.

**Figure 4.**
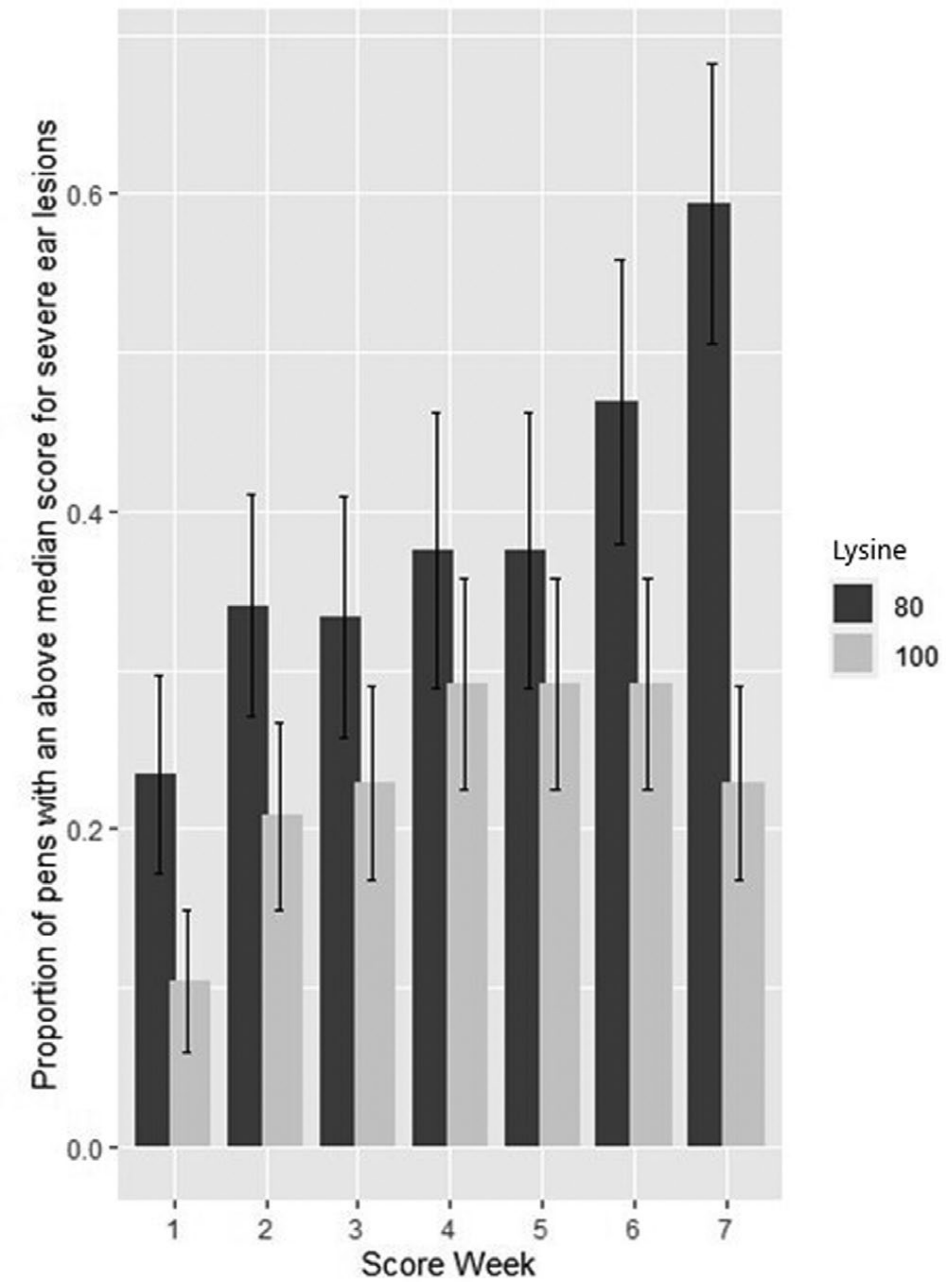
Effect of interaction between lysine (Lys) level and score week (*P* = 0.0104) on the number of pens showing severe ear lesions. The proportion of pens with an above-median score for severe ear lesions grouped by score week and lysine level; 100% Lys = diets with 100% PIC requirement at the midpoint of the growth phase (Lys 100) vs 80% Lys = diets with 80% PIC requirement at the midpoint of the growth phase (Lys 80). Forty-eight replicates per Lys level.

Lys and Trp:Lys interacted on the proportion of pens with an above-median score for severe ear lesions (*P* = 0.030). Pigs on the Lys 100 with Trp:Lys 0.185 and Lys 100 with Trp:Lys 0.160 treatments had lower numbers of pens with an above-median score for severe ear lesions compared to the other Lys and Trp:Lys combinations (Figure 3). Lys and score week also interacted with the number of pens showing severe ear lesions (*P* = 0.0104). Severe ear lesion incidence increased faster over time for Lys 80 than for Lys 100 pigs, resulting in a significant difference in the final score in week 7 (Figure 4). There was no effect of fill date or barn on the proportion of pens with an above-median score for severe ear lesions.

### Fight lesions

We found an effect of pig size (*P* < 0.001) and score week (*P* < 0.001) on fight lesions. Large pigs had fewer fight lesions (0.188 ± 0.0199) than small and medium pigs (0.372 ± 0.0247 and 0.326 ± 0.0239, respectively). Fight lesions were higher in score week 1 (0.389 ± 0.0288) than in score week 4 (0.198 ± 0.0235). Barn tended to affect fight lesion score (*P* = 0.055) but there was no significant effect of fill date, Lys, Trp:Lys, or the Lys × Trp:Lys interaction.

## Discussion

The main aim of this study was to explore the effect of dietary Lys and Trp, given at commercially relevant levels, on the expression of both positive and negative behaviors. We found no effect of diet on play behavior. This may be due to a combination of missing the majority of the pig’s play window (around 6 to 14 wk [Newberry et al., 1988]) and the housing conditions (slatted floors with no straw) not being conducive to play behaviors (Lyons et al., 1995). Other studies have also shown that nose-to-nose contact and allogrooming occurred infrequently in indoor-housed pigs (Camerlink et al., 2022). There is also evidence to suggest that social nosing is largely unrelated to negative social behaviors like tail biting (Camerlink and Turner, 2013) so it may be that the underlying mechanisms driving positive and negative social behaviors are different and strategies used to reduce one may not affect the other. Positive social behaviors are less well understood than negative social behaviors and are often harder to observe. We would benefit from further exploring the full behavioral repertoire of commercially housed pigs, looking at both positive and “neutral” social behaviors. It may be that “neutral” or “passive” social behaviors can better help us understand the social dynamics of groups and their welfare.

Previous work has shown that injuries from chronic aggression occur in varying amounts between pens (Desire et al., 2015). We found no dietary effect on fight lesions or aggressive behavior, suggesting that chronic aggression in stable social groups was unaffected by the amino acid levels in this population. The regrouping period, when fighting is most common, was not observed in this study and the effect of Lys and Trp levels on this form of aggression should be studied. As mentioned before, some studies found an effect of Trp on aggression at mixing (Shen et al., 2012b; Poletto et al., 2014) but this was when Trp was supplemented at high levels and these studies did not explore the effects of Lys at mixing.

We found there to be a significant effect of Lys on increased ear-biting behavior and an effect of the interaction between Lys and score week on ear lesions of any severity and specifically severe ear lesions. Lys is the first limiting amino acid in the majority of pig diets and so has been studied extensively in a production context but rarely in relation to the behavior and welfare of pigs. Srinongkote et al., (2003) found that diets fortified with a combination of Lys and arginine reduced plasma cortisol in pigs during transportation and Minussi et al., (2023) found that supplementing low protein diets with indispensable amino acids (L-Lysine, DL-Methionine, L-Threonine, L-Tryptophan, L-Valine, L-Isoleucine, L-Leucine and L-Histidine) reduced tail biting in pigs with intact tails. Our results, together with those of Minussi et al. (2023) and Srinongkote et al. (2003) strongly suggest an effect of Lys on pig behavior and further research is needed to explore this and to understand the mechanism of how Lys affects behavior. We speculate the increased ear biting could be due to nutritional deficiency and/or impaired immune response as a result of a Lys deficiency. Numerous studies have shown a link between low-protein diets and an increase in biting behaviors (Beattie et al., 2005; Meer et al., 2017). Studies have shown that pigs deficient in protein show increased attraction to blood (Fraser et al., 1991) and increased rooting behavior (Jensen et al., 1993). This combined attraction to blood and increased motivation to root may explain the increased ear biting.

The increased biting may also be due to low Lys levels affecting the health and immune response of pigs. Lys plays an important role in the immune response. Studies have shown that a lack of dietary Lys can impair an animal’s immune function, making them more susceptible to infections (Datta et al., 2001; Li et al., 2007). Studies in chickens estimated that the total cost of upregulating both the innate and adaptive immune system was between 7% and 10% of the animals total Lys requirements (Klasing, 2007), therefore a deficiency in Lys may limit the synthesis of immune response related proteins. Multiple studies have explored the link between disease and damaging behavior (review by Boyle et al., 2022) and the majority of the literature points towards an association between low health and negative social behaviors. Studies have linked internal inflammation to biting behavior (Czycholl et al., 2023) and uncovered a correlation between cytokine levels and social behavior (Munsterhjelm et al., 2017).

The effect of the Lys level and score week interaction may be due to a cumulative effect of Lys level. Prolonged nutritional deficiency and/or impaired immune response as a result of a Lys deficiency could result in more damaging tail/ear biting outbreaks, as pigs on low Lys diets may not have the capacity to recover and heal from bite lesions. As we have yet to understand the mechanisms by which Lys affects behavior, the long-term effects of low Lys on behavior likewise remain to be elucidated.

Score week could have influenced the severity of ear lesions for a number of reasons; external temperature and consequent changes to wind speed in the barn (van Putten, 1969; Geers et al., 1989), emptying of slurry pits and changes to gas levels in the barn (van Putten, 1969), coincidental removal of biters (biters tend to have lower growth rates (Beattie et al., 2005) so some of these pigs may have been removed from their pen due to illness/death, removing the biter/bitten pigs can reduce ear/tail biting (Zonderland et al., 2008)) and the onset and development of biting outbreaks, where severity and the number of lesions tend to increase over time. Pigs on low Lys diets may be less able to cope with these environmental changes as they may already be nutritionally/immune challenged.

Compared to ear biting, we found very little occurrence of tail biting during this study. This may have been due to the pigs’ tails being docked short (approximately 3 inches). Studies have shown that when tails are docked short, ear biting occurs more often than tail biting (Goossens et al., 2008). We recommend that the effect of commercially relevant levels of Lys and Trp on tail biting be studied in populations with a higher incidence of tail biting. The high incidences of ear lesions (as opposed to more commonly observed tail lesions) could also be linked to ear necrosis. Porcine ear necrosis (PEN) is a complex and not fully understood condition but may contribute to, or be exacerbated by, ear biting (Park et al., 2013). It may be the case that initially the ear lesions are caused by PEN and these lesions become bloody and then as mentioned above, attract pigs with a nutrient deficiency to the open wound causing an increase in biting behavior. Conversely, it may be that the act of ear biting introduces pathogens to the site that cause PEN. There are suggested infectious and non-infections factors affecting PEN, potential noninfectious factors involved in PEN include slatted floors with no straw, poor air quality, high stocking density and competition for drinker and feeder space (Park et al., 2013), factors commonly found in intensive systems. It is difficult to distinguish between ear lesions caused by biting and PEN. We did observe more ear biting behavior in pigs on the low Lys diets so can be relatively confident that the lesions we observed were largely due to ear biting. There are a small number of studies exploring the effect of Lys on the skin, feathers, and fur of other species (Kratzer and Vohra, 1956; Vohra and Kratzer, 1957). In kittens, Lys-deficient diets have been linked to crusted facial lesions (Larsen et al., 2010, 2014). It may be that low Lys effects the structural integrity of the pig’s skin, making the ears more susceptible to PEN or the flesh of the pigs more attractive to other pigs.

The significant interaction between Lys level and Trp:Lys on ear lesions suggests that the effect of Trp:Lys depends on Lys level. The results suggest that, in line with our hypothesis, sever ear lesions reduce at higher Trp:Lys for the Lys 80 treatment. However, the opposite was the case for the Lys 100 treatment, with the highest proportion of sever ear lesions seen with the highest Trp:Lys. Whilst it might be suggested that the latter could be due to faster-growing pigs having increased competition at the feeder or accelerated space reduction leading to increased ear biting, this was not supported by behavioral data (e.g., fight lesions). Therefore, it cannot be excluded that the increase in sever ear lesions in the Lys 100 with Trp:Lys 0.210 treatment pigs compared to their 0.185 and 0.160 counterparts were caused by factors other than negative social behaviors. From a production standpoint, previous studies have identified the optimum Trp:Lys to be between 0.17 (Susenbeth, 2006) to 0.203 (Xie et al., 2014). As this is the first trial to explore the effect of the Trp:Lys on negative social behaviors it is not clear what the optimum Trp:Lys is in relation to behavior and welfare. Further exploration of an optimal behavioral ratio is needed along with research to gain a better understanding of the interactive effect of the 2 amino acids on behavior, both positive and negative.

A key point in the trial was the removal of pigs from the Lys 80 with Trp:Lys 0.160 diet. As previously discussed, the pigs on this diet reached both their production (slowed growth rate) and welfare endpoints (as outlined in Materials and Methods). While the removal of these pigs was essential for ethical reasons, it left a gap in our research. We only had data from the first 2 feeding phases for these pigs, meaning we were unable to see how this treatment effected behavior over a longer period of time. We cannot speculate on the long-term effect of this treatment but we can say this diet had a serious negative impact on the production metrics and overall welfare of these pigs.

## Conclusion

In conclusion, the Lys content of feed can affect the social behavior of pigs, specifically ear biting, in commercial conditions. The link between dietary Lys and ear lesions and ear biting behavior requires further quantification and mechanistic exploration, and further research into the effects of Lys on tail biting should be conducted, preferably on pigs with undocked tails.

## Supplementary Material

skaf030_suppl_Supplementary_Appendix

## Abbreviations

Lys: lysine
PCA: principal component analysis
PC: principal component
PEN: porcine ear necrosis
SID: standardized ileal digestible
SRUC: Scotland’s Rural College
Trp: tryptophan
