# Supplementary material for: Exploring the effects of dietary lysine and tryptophan on the social behavior of pigs
Source: J Anim Sci. 2025 Feb 6;103:skaf030. doi: 10.1093/jas/skaf030 (PMC11912881; doi:10.1093/jas/skaf030)
Supplement: skaf030_suppl_Supplementary_Appendix [file skaf030_suppl_supplementary_appendix.docx]

**Appendix 1.**

Table A1. Ingredient composition of experimental diets (as-fed basis) for diets fed from 11.9 to 29.5 kg (Phase 1)

| **Ingredient name, %** | **T1^1^** | **T3** | **T4** | **T6** |
| --- | --- | --- | --- | --- |
| Corn | 54.79 | 54.83 | 60.64 | 60.66 |
| Distiller’s dried grain with solubles | 20.00 | 20.00 | 20.00 | 20.00 |
| Soybean meal | 21.00 | 21.00 | 15.73 | 15.77 |
| L-Lysine HCl | 0.64 | 0.64 | 0.49 | 0.49 |
| DL-Methionine | 0.22 | 0.22 | 0.12 | 0.12 |
| L-Threonine | 0.24 | 0.24 | 0.15 | 0.15 |
| L-Tryptophan | 0.07 | 0.01 | 0.05 | 0.00 |
| L-Isoleucine | 0.07 | 0.07 | 0.01 | 0.01 |
| L-Valine | 0.14 | 0.14 | 0.05 | 0.05 |
| Monocalcium phosphate | 0.50 | 0.50 | 0.57 | 0.57 |
| Limestone | 1.28 | 1.28 | 1.43 | 1.43 |
| Salt | 0.41 | 0.41 | 0.42 | 0.41 |
| Phytase (Axtra^®^ 2500)^2^ | 0.03 | 0.03 | 0.03 | 0.03 |
| Vitamin premix^3^ | 0.15 | 0.15 | 0.15 | 0.15 |
| Trace mineral premix^3^ | 0.15 | 0.15 | 0.15 | 0.15 |
| Corn oil | 0.30 | 0.33 | 0.00 | 0.00 |
| **Nutrient composition** | **T1** | **T3** | **T4** | **T6** |
| SID Lys, % (calculated) | 1.23 | 1.23 | 0.99 | 0.99 |
| SID Trp, % (calculated) | 0.26 | 0.20 | 0.21 | 0.16 |
| SID Trp:Lys (calculated) | 0.21 | 0.16 | 0.21 | 0.16 |
| Net energy, kcal/kg (calculated) | 2400 | 2400 | 2400 | 2400 |
| Dry matter, % | 86.18 | 86.44 | 86.40 | 85.62 |
| Crude protein, % | 20.14 | 20.57 | 18.38 | 18.43 |
| Lys, % | 1.29 | 1.30 | 1.04 | 1.08 |
| Trp, % | 0.26 | 0.22 | 0.20 | 0.18 |
| Neutral detergent fiber, % | 12.0 | 11.9 | 11.7 | 11.5 |
| Ether extract, % | 3.87 | 3.96 | 3.64 | 3.44 |
| Ash, % | 4.38 | 4.11 | 4.74 | 4.69 |
| Ca, % | 0.57 | 0.57 | 0.78 | 0.88 |
| P, % | 0.56 | 0.56 | 0.59 | 0.56 |
| STTD P, % (calculated) | 0.29 | 0.29 | 0.29 | 0.29 |

^1^T2 and T5 were prepared by mixing equal proportions of T1 and T3, and of T4 and T6, respectively.

^2^Phytase released 0.12% avP; 0.105% STTD P, and 0.24 total Ca.

^3^The combined vitamin and trace mineral premix provided per kg feed: 2,640 IU vitamin A, 880 IU vitamin D, 26.4 IU vitamin E, 2.75 mg vitamin K, 22 µg vitamin B12, 30.8 mg niacin, 17.6 mg pantothenic acid, 5.5 mg riboflavin, 242 mg/kg zinc (zinc sulfate), 242 mg/kg Fe (iron sulfate), 88 mg/kg Mn (manganese sulfate), 26.4 mg/kg Cu (copper sulfate), and 0.66 mg/kg Se (sodium selenite), and 450 phytase units per kilogram of diet.

**Appendix 1.**

Table A2. Ingredient composition of experimental diets (as-fed basis) for diets fed from 29.5 to 49.6 kg (Phase 2)

| **Ingredient name, %** | **T1^1^** | **T3** | **T4** | **T6** |
| --- | --- | --- | --- | --- |
| Corn | 49.25 | 49.28 | 56.48 | 56.57 |
| Distiller’s dried grain with solubles | 30.00 | 30.00 | 30.00 | 30.00 |
| Soybean meal | 17.14 | 17.14 | 10.64 | 10.64 |
| L-Lysine HCl | 0.56 | 0.56 | 0.47 | 0.47 |
| DL-Methionine | 0.13 | 0.13 | 0.05 | 0.05 |
| L-Threonine | 0.17 | 0.17 | 0.11 | 0.11 |
| L-Tryptophan | 0.06 | 0.00 | 0.04 | 0.00 |
| L-Isoleucine | 0.02 | 0.02 | 0.00 | 0.00 |
| L-Valine | 0.09 | 0.09 | 0.03 | 0.03 |
| Monocalcium phosphate | 0.24 | 0.24 | 0.32 | 0.32 |
| Limestone | 1.27 | 1.27 | 1.32 | 1.29 |
| Salt | 0.34 | 0.34 | 0.35 | 0.35 |
| Phytase (Axtra^®^ 2500)^2^ | 0.03 | 0.03 | 0.03 | 0.03 |
| Vitamin-mineral premix^3^ | 0.15 | 0.15 | 0.15 | 0.15 |
| Corn oil | 0.56 | 0.58 | 0.00 | 0.00 |
| **Nutrient composition** | **T1** | **T3** | **T4** | **T6** |
| SID Lys, % (calculated) | 1.11 | 1.11 | 0.89 | 0.89 |
| SID Trp, % (calculated) | 0.23 | 0.18 | 0.19 | 0.14 |
| SID Trp:Lys (calculated) | 0.21 | 0.16 | 0.21 | 0.16 |
| Net energy, kcal/kg (calculated) | 2418 | 2418 | 2418 | 2418 |
| Dry matter, % | 86.21 | 86.44 | 86.76 | 86.36 |
| Crude protein, % | 21.03 | 20.99 | 18.67 | 18.56 |
| Lys, % | 1.20 | 1.19 | 1.01 | 0.99 |
| Trp, % | 0.24 | 0.20 | 0.19 | 0.16 |
| Neutral detergent fiber, % | 13.1 | 12.5 | 12.7 | 12.8 |
| Ether extract, % | 4.70 | 4.65 | 4.47 | 4.08 |
| Ash, % | 4.50 | 4.48 | 4.35 | 4.27 |
| Ca, % | 0.61 | 0.65 | 0.64 | 0.60 |
| P, % | 0.49 | 0.50 | 0.51 | 0.50 |
| STTD P, % (calculated) | 0.26 | 0.26 | 0.26 | 0.26 |

^1^T2 and T5 were prepared by mixing equal proportions of T1 and T3, and of T4 and T6, respectively.

^2,3^ See Table A1

**Appendix 1.**

Table A3. Ingredient composition of experimental diets (as-fed basis) for diets fed from 49.6 to 62.6 kg (Phase 3)

| **Ingredient name, %** | **T1^1^** | **T3** | **T4** | **T5** |
| --- | --- | --- | --- | --- |
| Corn | 54.37 | 54.40 | 60.47 | 60.51 |
| Distiller’s dried grain with solubles | 30.00 | 30.00 | 30.00 | 30.00 |
| Soybean meal | 12.62 | 12.62 | 7.04 | 7.04 |
| L-Lysine HCl | 0.50 | 0.50 | 0.42 | 0.42 |
| DL-Methionine | 0.08 | 0.08 | 0.01 | 0.01 |
| L-Threonine | 0.13 | 0.13 | 0.08 | 0.08 |
| L-Tryptophan | 0.05 | 0.00 | 0.04 | 0.02 |
| L-Isoleucine | 0.01 | 0.01 | 0.00 | 0.00 |
| L-Valine | 0.06 | 0.06 | 0.00 | 0.00 |
| Monocalcium phosphate | 0.03 | 0.03 | 0.10 | 0.10 |
| Limestone | 1.19 | 1.19 | 1.30 | 1.28 |
| Salt | 0.35 | 0.35 | 0.36 | 0.36 |
| Phytase (Axtra^®^ 2500)^2^ | 0.03 | 0.03 | 0.03 | 0.03 |
| Vitamin-mineral premix^3^ | 0.15 | 0.15 | 0.15 | 0.15 |
| Corn oil | 0.44 | 0.47 | 0.00 | 0.00 |
| **Nutrient composition** | **T1** | **T3** | **T4** | **T5** |
| SID Lys, % (calculated) | 0.98 | 0.98 | 0.77 | 0.77 |
| SID Trp, % (calculated) | 0.20 | 0.15 | 0.16 | 0.12 |
| SID Trp:Lys (calculated) | 0.21 | 0.16 | 0.21 | 0.16 |
| Net energy, kcal/kg (calculated) | 2442 | 2442 | 2442 | 2442 |
| Dry matter, % | 86.90 | 87.02 | 86.99 | 86.23 |
| Crude protein, % | 18.95 | 19.18 | 17.13 | 17.00 |
| Lys, % | 1.02 | 1.08 | 0.96 | 0.86 |
| Trp, % | 0.21 | 0.19 | 0.18 | 0.19 |
| Neutral detergent fiber, % | 12.8 | 13.3 | 12.7 | 13.7 |
| Ether extract, % | 4.24 | 4.34 | 3.73 | 3.87 |
| Ash, % | 4.24 | 4.08 | 4.06 | 3.74 |
| Ca, % | 0.62 | 0.62 | 0.57 | 0.54 |
| P, % | 0.47 | 0.49 | 0.52 | 0.52 |
| STTD P, % (calculated) | 0.21 | 0.21 | 0.21 | 0.21 |

^1^T2 was prepared by mixing equal proportions of T1 and T3.

^2,3^ See Table A1

**Appendix 1.**

Table A4. Ingredient composition of experimental diets (as-fed basis) for diets fed from 62.6 to 80.3 kg (Phase 4)

| **Ingredient name, %** | **T1^1^** | **T3** | **T4** | **T5** |
| --- | --- | --- | --- | --- |
| Corn | 70.53 | 70.71 | 72.87 | 72.95 |
| Distiller’s dried grain with solubles | 15.00 | 15.00 | 15.00 | 15.00 |
| Soybean meal | 12.03 | 11.87 | 9.82 | 9.76 |
| L-Lysine HCl | 0.40 | 0.41 | 0.26 | 0.27 |
| DL-Methionine | 0.07 | 0.08 | 0.00 | 0.00 |
| L-Threonine | 0.12 | 0.12 | 0.04 | 0.04 |
| L-Tryptophan | 0.04 | 0.00 | 0.02 | 0.00 |
| L-Isoleucine | 0.02 | 0.02 | 0.00 | 0.00 |
| L-Valine | 0.08 | 0.08 | 0.00 | 0.00 |
| Monocalcium phosphate | 0.04 | 0.04 | 0.07 | 0.07 |
| Limestone | 1.03 | 1.04 | 1.28 | 1.28 |
| Salt | 0.46 | 0.46 | 0.46 | 0.46 |
| Phytase (Axtra^®^ 2500)^2^ | 0.03 | 0.03 | 0.03 | 0.03 |
| Vitamin-mineral premix^3^ | 0.15 | 0.15 | 0.15 | 0.15 |
| Corn oil | 0.00 | 0.00 | 0.00 | 0.00 |
| **Nutrient composition** | **T1** | **T3** | **T4** | **T5** |
| SID Lys, % (calculated) | 0.82 | 0.82 | 0.65 | 0.65 |
| SID Trp, % (calculated) | 0.17 | 0.13 | 0.14 | 0.10 |
| SID Trp:Lys (calculated) | 0.21 | 0.16 | 0.21 | 0.185 |
| Net energy, kcal/kg (calculated) | 2459 | 2459 | 2459 | 2459. |
| Dry matter, % | 85.38 | 85.91 | 85.80 | 85.52 |
| Crude protein, % | 15.61 | 15.32 | 14.40 | 14.09 |
| Lys, % | 0.88 | 0.84 | 0.67 | 0.70 |
| Trp, % | 0.17 | 0.14 | 0.15 | 0.14 |
| Neutral detergent fiber, % | 9.8 | 9.1 | 10.0 | 9.4 |
| Ether extract, % | 3.96 | 3.94 | 3.83 | 3.70 |
| Ash, % | 3.07 | 3.23 | 3.10 | 3.26 |
| Ca, % | 0.40 | 0.50 | 0.44 | 0.50 |
| P, % | 0.35 | 0.36 | 0.36 | 0.37 |
| STTD P, % (calculated) | 0.17 | 0.17 | 0.17 | 0.17 |

^1^T2 was prepared by mixing equal proportions of T1 and T3.

^2,3^ See Table A1

**Appendix 1.**

Table A5. Ingredient composition of experimental diets (as-fed basis) for diets fed from 80.3 to 103.3 kg (Phase 5)

| **Ingredient name, %** | **T1^1^** | **T3** | **T4** | **T5** |
| --- | --- | --- | --- | --- |
| Corn | 79.02 | 79.05 | 79.57 | 79.58 |
| Distiller’s dried grain with solubles | 9.75 | 9.75 | 9.75 | 9.74 |
| Soybean meal | 8.66 | 8.65 | 8.51 | 8.51 |
| L-Lysine HCl | 0.40 | 0.40 | 0.22 | 0.22 |
| DL-Methionine | 0.07 | 0.07 | 0.00 | 0.00 |
| L-Threonine | 0.12 | 0.12 | 0.03 | 0.03 |
| L-Tryptophan | 0.04 | 0.01 | 0.01 | 0.00 |
| L-Isoleucine | 0.04 | 0.04 | 0.00 | 0.00 |
| L-Valine | 0.09 | 0.09 | 0.00 | 0.00 |
| Monocalcium phosphate | 0.00 | 0.00 | 0.00 | 0.00 |
| Limestone | 0.94 | 0.94 | 0.95 | 0.95 |
| Salt | 0.50 | 0.50 | 0.50 | 0.50 |
| Phytase (Axtra^®^ 2500)^2^ | 0.03 | 0.03 | 0.03 | 0.03 |
| Vitamin-mineral premix^3^ | 0.15 | 0.15 | 0.15 | 0.15 |
| Corn oil | 0.19 | 0.20 | 0.29 | 0.30 |
| **Nutrient composition** | **T1** | **T3** | **T4** | **T5** |
| SID Lys, % (calculated) | 0.72 | 0.72 | 0.57 | 0.57 |
| SID Trp, % (calculated) | 0.15 | 0.11 | 0.12 | 0.11 |
| SID Trp:Lys (calculated) | 0.21 | 0.16 | 0.21 | 0.185 |
| Net energy, kcal/kg (calculated) | 2502 | 2502 | 2502 | 2502 |
| Dry matter, % | 84.92 | 84.93 | 85.34 | 85.64 |
| Crude protein, % | 13.86 | 13.51 | 13.79 | 13.82 |
| Lys, % | 0.80 | 0.75 | 0.69 | 0.67 |
| Trp, % | 0.15 | 0.13 | 0.14 | 0.14 |
| Neutral detergent fiber, % | 9.4 | 8.0 | 8.3 | 8.6 |
| Ether extract, % | 4.02 | 3.66 | 5.21 | 4.02 |
| Ash, % | 3.03 | 3.11 | 3.00 | 2.96 |
| Ca, % | 0.45 | 0.46 | 0.50 | 0.34 |
| P, % | 0.30 | 0.29 | 0.29 | 0.29 |
| STTD P, % (calculated) | 0.14 | 0.14 | 0.14 | 0.14 |

^1^T2 was prepared by mixing equal proportions of T1 and T3.

^2,3^ See Table A1
